# Genomic Identification of the TOR Signaling Pathway as a Target of the Plant Alkaloid Antofine in the Phytopathogen Fusarium graminearum

**DOI:** 10.1128/mBio.00792-19

**Published:** 2019-06-11

**Authors:** Christopher Mogg, Christopher Bonner, Li Wang, Johann Schernthaner, Myron Smith, Darrell Desveaux, Rajagopal Subramaniam

**Affiliations:** a Donnelly Centre for Cellular and Biomolecular Research, University of Toronto, Toronto, Ontario, Canada; b Agriculture and Agri-Food Canada, Ottawa, Ontario, Canada; c Department of Biology, Carleton University, Ottawa, Ontario, Canada; d Department of Cell and Systems Biology, University of Toronto, Toronto, Ontario, Canada; University of British Columbia

**Keywords:** *Fusarium*, drug targets, rapamycin

## Abstract

Antofine, a phenanthroindolizidine alkaloid, is a bioactive natural product isolated from milkweeds that exhibits numerous biological activities, including anticancer, antimicrobial, antiviral, and anti-inflammatory properties. However, the direct targets and mode of action of antofine have not been determined. In this report, we show that antofine displays antifungal properties against the phytopathogen Fusarium graminearum, the cause of *Fusarium* head blight disease (FHB). FHB does devastating damage to agriculture, causing billions of dollars in economic losses annually. We therefore sought to understand the mode of action of antofine in F. graminearum using insights from yeast chemical genomic screens. We used haploinsufficiency profiling (HIP) to identify putative targets of antofine in yeast and identified three candidate targets, two of which had homologs in F. graminearum. The *Fusarium* homologues of two targets, glutamate dehydrogenase (*Fg*GDH) and resistance to rapamycin deletion 2 (*Fg*RRD2), can bind antofine. Of the two genes, only the *Fgrrd2* knockout displayed a loss of virulence in wheat, indicating that RRD2 is an antivirulence target of antofine in F. graminearum. Mechanistically, we demonstrate that antofine disrupts the interaction between *Fg*RRD2 and *Fg*Tap42, which is part of the Tap42-phosphatase complex in the target of rapamycin (TOR) signaling pathway, a central regulator of cell growth in eukaryotes and a pathway of extensive study for controlling numerous pathologies.

## Abstract (precis)

*Fusarium* head blight caused by the fungal pathogen Fusarium graminearum is a devastating disease of cereal crops worldwide, with limited effective chemical treatments available. Here we show that the natural alkaloid compound antofine can inhibit fusarium head blight in wheat. Using yeast genomic screening, we identified the TOR pathway component RRD2 as a target of antofine that is also required for F. graminearum pathogenicity.

## INTRODUCTION

Antofine belongs to the phenanthroindolizidine class of alkaloids that are produced by relatives of the milkweed family (*Apocynaceae* subfamily *Asclepiadoideae*), which include *Vincetoxicum* spp., as well as members of the related genus *Tylophora* (1). These compounds have received significant attention as candidate anticancer agents, which promote apoptosis in cancer cell lines by inhibiting nuclear factor-kappa B (NF-κB) (2). Antofine has also been shown to suppress DNA and suppress cell cycle arrest as well as endosomal signaling (3, 4). More recently, the compound has been shown to inhibit angiogenesis in endothelial cells (5). Specifically, vascular endothelial growth factor (VEGF), which stimulates angiogenesis through the action of protein kinase B or AKT/mTOR signaling pathways, is inhibited by antofine via an unknown mechanism (5).

Antofine has also been shown to inhibit growth of a variety of microorganisms, including two strains of the phytopathogen Fusarium graminearum (6), which causes *Fusarium* head blight (FHB) disease in small-grain cereals, resulting in low-yield, low-quality, mycotoxin-contaminated grain, which poses a serious threat to food safety and the economy (7). Due to the ubiquitous global distribution of F. graminearum, most agronomic practices aimed at controlling FHB, including chemical control, offer only moderate control and do not eliminate the proliferation of the disease. Only a few active ingredients have been registered to suppress F. graminearum as a foliar or seed treatment in cereals (8). Most of these compounds belong to the triazole group of fungicides and include tebuconazole, triticonazole, difenoconazole, and ipconazole. The efficacy of these fungicides can vary between *Fusarium* species, and moreover, emergence of fungicide-resistant strains is compelling stakeholders to develop new strategies to combat FHB disease (9, 10).

Although ubiquitous in eukaryotes, only recently has the TOR signaling pathway been documented to play a role in F. graminearum development and pathogenesis (11). Inhibition of TOR signaling by rapamycin, which binds Fkbp12 to inhibit Tor kinase and the downstream Tap42 protein phosphatase complex, reduces both vegetative growth and conidial germination, leading to reduced pathogenicity (11). In addition, two peptidyl-proly *cis*/*trans*-isomerases, RRD1 (FGSG_09229) and RRD2 (FGSG_01092), which regulate the activity of type 2A phosphatases of the TOR pathway and confer resistance to rapamycin when mutated (12), have overlapping functions in mycotoxin production and virulence, as well as growth and reproduction (13). However, the severity of growth defects and asexual spore production are more pronounced in the F. graminearum *rrd2* (*Fgrrd2*) mutant than *Fgrrd1* (13). In addition, *Fg*RRD2, but not *Fg*RRD1, modulates sensitivity to the phenylpyrrole fungicide fludioxonil and plays a dominant role in regulation of the high-osmolarity glycerol (HOG) pathway in F. graminearum (13).

In this report, we use yeast haploinsufficiency profiling (HIP) to identify potential targets of antofine. We show that RRD2 is a target of antofine in F. graminearum. Additionally, we show that binding of antofine to RRD2 leads to the disruption of the Tap42 phosphatase complex, providing a potential mechanism for disruption of the TOR signaling pathway.

## RESULTS AND DISCUSSION

**FIG 1:**
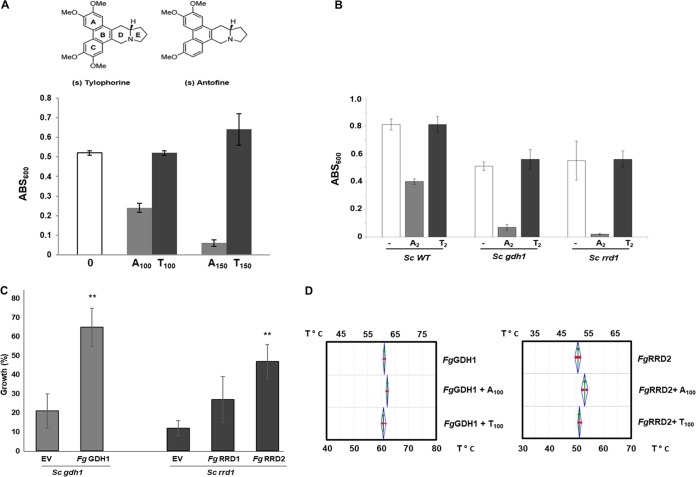
Antofine inhibits growth of F. graminearum and targets GDH1 and RRD1. (A). Wild-type F. graminearum (*Fg*) spores were coincubated with 100 μg/ml of antofine (A_100_), tylophorine (T_100_), or solvent control (−), and mycelial growth was monitored by absorbance at 600 nm (ABS_600_). Results are representative of five biological replicates. The chemical structures of tylophorine and antofine are shown. (B) Growth of wild-type S. cerevisiae (*Sc WT*) or *Scgdh1* and *Scrrd1* mutants in the presence of 2 μg/ml antofine (A_2_), tylophorine (T_2_), or solvent control (−) was monitored by absorbance at 600 nm (ABS_600_). Values represent the average of three technical replicates with standard deviation. The results are representative of four biological replicates. (C) F. graminearum GDH1 and RRD2 complement antofine hypersensitivity to the *Scgdh1* and *Scrrd1* mutants, respectively. *Fg GDH1* (FGSG_07174) was expressed in the *Scgdh1* mutant, and *FgRRD1* (FGSG_09229) and *Fg*RRD2 (FGSG_01092) were expressed in the S. cerevisiae *Scrrd1* mutant. Empty vectors (EVs) were used as controls. Expression of *FgGDH1* and *FgRRD1* was induced by galactose and was monitored in the absence and presence of 2 μg/ml antofine. Values represent average percentage of growth with respect to growth in the absence of antofine of three technical replicates with standard deviation. (D) Thermal shift assays using 1 mg/ml of purified *Fg*GDH1 and *Fg*RRD2 proteins in the absence and presence of antofine (A_100_), and tylophorine (T_100_) at 100 μg/ml. Results are representative of two independent biological replicates with four technical replicates in each experiment.

We purified antofine from *Vincetoxicum* to homogeneity as previously described (see Fig. S1A in the supplemental material) (6). After establishing a standard growth curve based on spore concentrations (Fig. S1B) and a linear relationship between mycelial growth and weight (Fig. S1C), we showed that antofine suppressed F. graminearum germination and growth in a dose-dependent manner with a 50% inhibitory concentration (IC_50_) of ∼100 μg/ml (see Fig. S2A in the supplemental material). Tylophorine is structurally related to antofine, and both compounds display similar activities, including growth inhibition of many cancer cell lines by blocking S-phase transition and arresting HepG2, HONE-1, and NUCG-3 carcinoma cells at the G_1_ phase (14). However, the suppression of F. graminearum germination and growth (as well as yeast growth [Fig. 1B]) was specific to antofine, and we therefore used tylophorine as a biologically inactive negative control (Fig. 1A) (15). In addition to suppressing *in vitro* growth, antofine decreased FHB symptoms on wheat heads when coinoculated with F. graminearum spores (Fig. S2B). It should be noted that at the antofine concentrations tested, antofine alone did not produce any observable deleterious effects such as kernel weight (data not shown).

10.1128/mBio.00792-19.1 FIG S1 Purification of antofine and growth properties of F. graminearum. (A) Purity of antofine confirmed by NMR (30). Antofine purified by TLC was subjected to proton NMR spectroscopy (CDCl_3_, 400 MHz): peaks represented by δ 7.90 (s, 1H), 7.89 (d, *J* = 2.6 Hz, 1H), 7.81 (d, *J* = 9.1 Hz, 1H), 7.30 (s, 1H), 7.20 (dd, *J* = 9.0, 2.5 Hz, 1H), 4.68 (d, *J* = 14.9 Hz, 1H), 4.10 (s, 3H), 4.06 (s, 3H), 4.01 (s, 3H), 3.68 (br d, *J* = 15.0 Hz, 1H), 3.45 (td, *J* = 8.5, 1.9 Hz, 1H), 3.33 (dd, *J* = 15.8, 2.4 Hz, 1H), 2.91 to 2.85 (m, 1H), 2.51 to 2.40 (m, 2H), 2.27 to 2.19 (m, 1H), 2.09 to 1.97 (m, 1H), 1.96 to 1.86 (m, 1H), and 1.81 to 1.71 (m, 1H). (B) Growth curve of F. graminearum spores grown in GYEP medium. In a volume of 200 μl, the medium was inoculated with 250 spores (orange line), 500 spores (blue line), and 1,000 spores (yellow line) in a 96-well plate, and spore germination and mycelial growth were monitored by absorbance at 600 nm (ABS_600_) over a period of 48 h (Hr). (C) A linear relationship between mycelial growth and weight. In a volume of 200 μl, the GYEP medium was inoculated with 500 spores in a 96-well plate. At various time points between 24 and 48 h, absorbance was measured and mycelia from 96 wells between were pooled, vacuum dried, and weighed. The values represent average of 96 wells with standard error. Download FIG S1, TIF file, 0.7 MB. © Crown copyright 2019. 2019 Crown This content is distributed under the terms of the Creative Commons Attribution 4.0 International license.

10.1128/mBio.00792-19.2 FIG S2 Antofine inhibits growth in a dose-dependent manner and reduces infection on wheat heads. (A) Wild-type F. graminearum spores were coincubated with antofine at 50, 100, and 200 μg/ml, and mycelial growth was monitored by absorbance at wavelength 600 (ABS_600_). This experiment was repeated at least four times. (B) Pathology tests were performed on a susceptible variety of wheat (Roblin), and disease progression was assessed over days postinfection (DPI). This experiment was repeated twice with similar outcomes. Images of wheat spikelets infected with wild-type F. graminearum spores and spores pretreated with antofine at 100 μg/ml (100) are shown. Download FIG S2, TIF file, 0.7 MB. © Crown copyright 2019. 2019 Crown This content is distributed under the terms of the Creative Commons Attribution 4.0 International license.

In order to identify potential targets of antofine, we used haploinsufficiency profiling (HIP) in yeast (16). HIP exploits drug-induced haploinsufficiency, as measured by growth defects resulting from deletion of one copy of a drug target gene, and has been used to identify the targets of small molecules, including fungicides (16). After establishing a sublethal concentration (2 μg/ml) of antofine (see Fig. S3A in the supplemental material), we conducted an HIP screen on ∼6,000 yeast heterozygous strains and identified three heterozygous strains that were hypersensitive to antofine. While wild-type (WT) yeast growth was inhibited by 2.5% at 2.0 μg/ml of antofine (∼4 μM), the 3 heterozygous mutants YOR375C, YBR095C, and YIL153W displayed 23, 15.5, and 12.5% growth inhibition, respectively (Fig. S3B). The Saccharomyces cerevisiae glutamate dehydrogenase gene (*ScGDH1*; YOR375C) has a homologue in F. graminearum (FGSG_07174) with 73% identity, and the *ScRRD1* gene (resistant to rapamycin deletion 1; YIL153W) has two homologues in F. graminearum, FGSG_09229 (*FgRRD1*) and FGSG_01092 (*FgRRD2*), with identities of 38 and 32%, respectively (13). The third target identified in yeast YBR095C showed a weak homology (18% identity) with F. graminearum gene FGSG_08612, and the homology between these proteins was not contiguous. Therefore, we pursued *FgGDH1*, *FgRRD1*, and *FgRRD2* for further characterization as putative targets of antofine. Note that both *Scgdh1* and *Scrrd1* mutant strains displayed no hypersensitivity to the negative-control tylophorine (Fig. 1B).

10.1128/mBio.00792-19.3 FIG S3 Haploinsufficiency profiling (HIP) for antofine targets in yeast. (A) Dosage inhibition of S. cerevisiae growth by antofine. Wild-type S. cerevisiae was assessed for growth by absorbance at wavelength 600 (ABS_600_) in the presence of antofine at 0, 1, 2, and 5 μg/ml. The IC_50_ was estimated to be ∼2 μg/ml of antofine. (B) Growth of wild-type yeast (S. cerevisiae [*Sc*]) and putative antofine targets identified by haploinsufficiency profiling (HIP), YBR095C, YOR375C, and YIL153W, was assessed in antofine at 2.0 μg/ml. Growth inhibition relative to wild-type S. cerevisiae is shown. Download FIG S3, TIF file, 0.5 MB. © Crown copyright 2019. 2019 Crown This content is distributed under the terms of the Creative Commons Attribution 4.0 International license.

To determine if F. graminearum genes could complement yeast mutants, we expressed *Fg*RRD1 (FGSG_09229) and *Fg*RRD2 (FGSG_01092) in the S. cerevisiae YIL153 mutant strain and expressed *FgGDH1* (FGSG_07174) in the S. cerevisiae YOR375C mutant strain. *Fg*GDH1 was able to partially complement the antofine sensitivity of the *Scgdh1* mutant strain (Fig. 1C). Interestingly, *Fg*RRD2 (*P* < 0.01) but not *Fg*RRD1 was able to partially rescue antofine sensitivity in the *Scrrd1* mutant strain and was therefore chosen for further analysis (Fig. 1C). To test if *Fg*GDH1 and *Fg*RRD2 are direct targets of antofine, both genes were cloned and expressed, and the purified protein (see Fig. S4 in the supplemental material) was used in thermal shift assays (TSAs) (17, 18). TSA determines a shift in the melting temperature (*T_m_*) of a protein upon binding to a ligand, measured by changes in light scattering or fluorescence. The *T_m_* of *Fg*GDH shifted from 61°C to 62.4°C in the presence of 100 μg/ml of antofine (Fig. 1D; +A_100_); however, in the presence of 100 μg/ml of tylophorine, the *T_m_* did not shift relative to *Fg*GDH alone (Fig. 1D; T_100_). The TSA also showed that the *T_m_* of purified *Fg*RRD2 shifted from 51°C to 54°C (Fig. 1D; A_100_) and similar to *Fg*GDH, the shift with tylophorine was negligible compared to that with the protein alone (Fig. 1D; T_100_). The TSA results indicate that antofine binds both *Fg*GDH and *Fg*RRD2 proteins.

10.1128/mBio.00792-19.4 FIG S4 Expression and purification of *Fg*GDH1 and *Fg*RRD2 from S. cerevisiae. *Fg*GDH1 and *Fg*RRD2 were cloned into pYES-DEST52 vector and transformed into the corresponding yeast knockout strains. The genes were induced by galactose, and proteins were purified using Ni-based affinity chromatography. Purity was assessed by SDS-PAGE, and proteins were stained with Coomassie blue stain. Lanes 1 and 6 represent crude protein extract after induction. Lane 2 represents residual protein after extraction. Lanes 3 and 7 represent proteins eluted with 20 mM imidazole; lane 4 represents an additional wash step with 50 mM imidazole. Lanes 5 and 8 represent the final elution step with 500 mM imidazole. Download FIG S4, TIF file, 0.8 MB. © Crown copyright 2019. 2019 Crown This content is distributed under the terms of the Creative Commons Attribution 4.0 International license.

**FIG 2:**
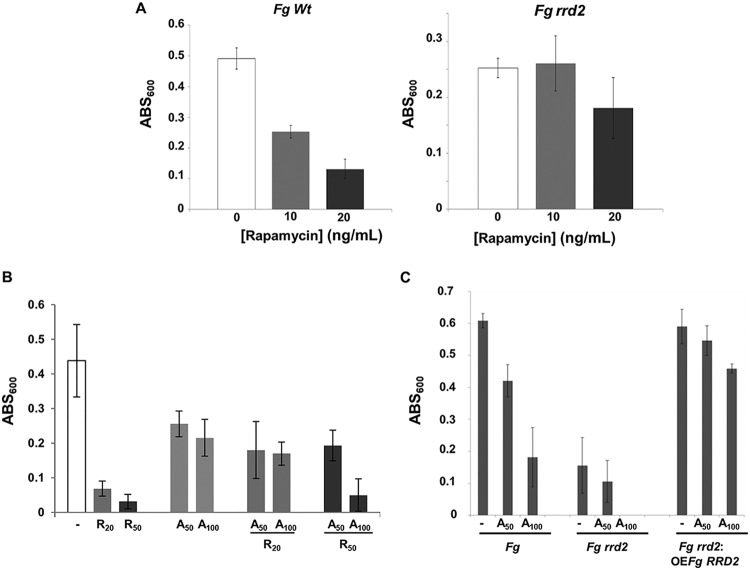
Antofine phenocopies the *rrd2* mutation in F. graminearum by suppressing rapamycin toxicity. (A) Growth of the F. graminearum *RRD2* mutant (*Fgrrd2*) is insensitive to rapamycin inhibition. Growth inhibition of wild-type (*Fg Wt*) or RRD2 mutant (*Fg rrd2)* F. graminearum spores coincubated with 10 and 20 ng/ml of rapamycin was monitored by absorbance at 600 nm (ABS_600_). (B) Growth inhibition of wild-type F. graminearum by rapamycin is suppressed by antofine (A_50_ and A_100_). Growth (optical density at 600 nm [OD_600_]) of wild-type F. graminearum spores was monitored in the presence of rapamycin at concentrations of 20 or 50 ng/ml (R_20_ and R_50_) alone or in combination with 50 or 100 μg/ml of antofine (A_50_ and A_100_). (C) Overexpression of RRD2 suppresses antofine toxicity. The mycelial growth of wild-type (*Fg*), *FgRRD2* mutant (*Fg rrd2*), and overexpressor of *RRD2* (*Fg rrd2*:OE*Fg RRD2*) *F. graminearum* strains was monitored by absorbance at 600 nm (ABS_600_) in the absence (−) and in the presence of 50 and 100 ng/ml of antofine (A_50_ and A_100_). Values represent the average from three technical replicates with standard deviation. Results are representative of four independent biological replicates.

Since antofine inhibited FHB symptoms on wheat (Fig. S2B), we tested whether both antofine targets were associated with fusarium pathogenicity. We deleted *GDH1* and *RRD2* in F. graminearum and performed pathology tests on the susceptible wheat variety Roblin. The deletion of *FgGDH1* did not affect the pathogenicity, suggesting that it is not the antofine target responsible for F. graminearum virulence (see Fig. S5A in the supplemental material). In contrast, targeted deletion of *FgRRD2* significantly affected the virulence of F. graminearum (Fig. S5B). After 15 days postinfection (dpi), the entire wheat head was bleached after inoculation with wild-type F. graminearum spores (Fig. S5B; *Fg*), while no such effect was visible after inoculation with the mutant strain (Fig. S5B; *Fg rrd2*). It is likely that growth defects and its inability to produce the mycotoxin deoxynivalenol significantly contributes to the virulence defects (13). Overexpression of *FgRRD2* in the *Fgrrd2* mutant strain (*Fgrrd1*::OE*FgRRD2*) not only restored F. graminearum virulence (Fig. S5b; *Fg rrd2*:OE*Fg RRD2*) but also counteracted the effect of antofine (Fig. 2C). These results suggest that although antofine appears to have multiple targets, *Fg*RRD2 is the target responsible for virulence inhibition in F. graminearum (13).

10.1128/mBio.00792-19.5 FIG S5 *Fg*RRD2 contributes to virulence. Pathology tests were performed on a susceptible variety of wheat (Roblin) with spores from wild-type F. graminearum (*Fg*), the *Fgrrd2* mutant, and the *Fgrrd2* mutant overexpressing RRD2 (*Fg rrd2*:OE*Fg RRD2*). Disease progression in pathology assays was quantified as indicated (days postinfection [DPI]) (34). All the pathology experiments were performed twice, and values represent averages with standard error from 10 infected wheat heads. The inset shows images of wheat spikelets infected with wild-type (*Fg*) and mutant (*Fg rrd2*) spores. Download FIG S5, TIF file, 0.6 MB. © Crown copyright 2019. 2019 Crown This content is distributed under the terms of the Creative Commons Attribution 4.0 International license.

The haploid yeast *rrd1* mutant and mutants of the two homologues in the F. graminearum strain PH1, *Fgrrd1* and *Fgrrd2*, have been shown to be resistant to the inhibitory effects of rapamycin (12, 13). We confirmed this phenotype for the *Fgrrd2* mutant in our F. graminearum strain (DAOM 233423 [Fig. 2]). Wild-type F. graminearum spores pretreated with rapamycin at 10 and 20 ng/ml decreased in growth by 50 and 80%, respectively, relative to untreated spores (Fig. 2A; *Fg Wt*). However, no such growth inhibition was observed with the *Fgrrd2* mutant strain exposed to rapamycin (Fig. 2A; *Fg rrd2*). We therefore hypothesized that if antofine antagonizes RRD2, it should chemically phenocopy the rapamycin insensitivity of the *Fgrrd2* mutant. In support of this, growth of wild-type F. graminearum spores was monitored in the presence of sublethal doses of antofine (50 and 100 μg/ml [A_50_ and A_100_, respectively]) and inhibitory concentrations of rapamycin (20 and 50 ng/ml [R_20_ and R_50_, respectively]) (Fig. 2B). We observed that the inhibitory effects of rapamycin at both doses were suppressed by antofine at 50 μg/ml (Fig. 2B). Antofine at 100 μg/ml suppressed the inhibitory effect of rapamycin at the lower concentration (20 ng/ml) but not at the higher concentration (50 ng/ml), likely due to synergism between antofine and rapamycin toxicity at higher concentrations. Altogether, the results strongly support RRD2 as a direct target of antofine.

RRD is a component of the target of the rapamycin signaling pathway, which is evolutionarily conserved and important for cell growth in eukaryotes (19). In yeast, the rapamycin-sensitive Tor complex 1 (TORC1), in addition to promoting anabolic processes, leads to suppression of stress response pathways (20). This dual role makes this pathway an extremely important subject area of study since its disruption either through mutation or through chemical inhibition can influence numerous diseases, including cancer and diabetes (21). TORC1 mediates these pathways through regulation of a set of serine/threonine protein phosphatases, including Sit4, protein phosphatase 2A (PP2A), and Ppg1, which together form the phosphatase-associating protein Tap42 protein complex in a nutrient- and Tor-dependent manner (22). The current model suggests that TORC1 is associated with the Tap42-phosphatase complexes, and upon nutrient stress or rapamycin treatment, the Tap42-phosphatase complex is released from TORC1 (22). This event disassembles the Tap42-phosphatase complex, leading to the activation of the phosphatases and inhibition of Tor activity (19). Therefore, as a positive regulator of phosphatases, Tap42 represents an important regulator of the TOR signaling pathway.

**FIG 3:**
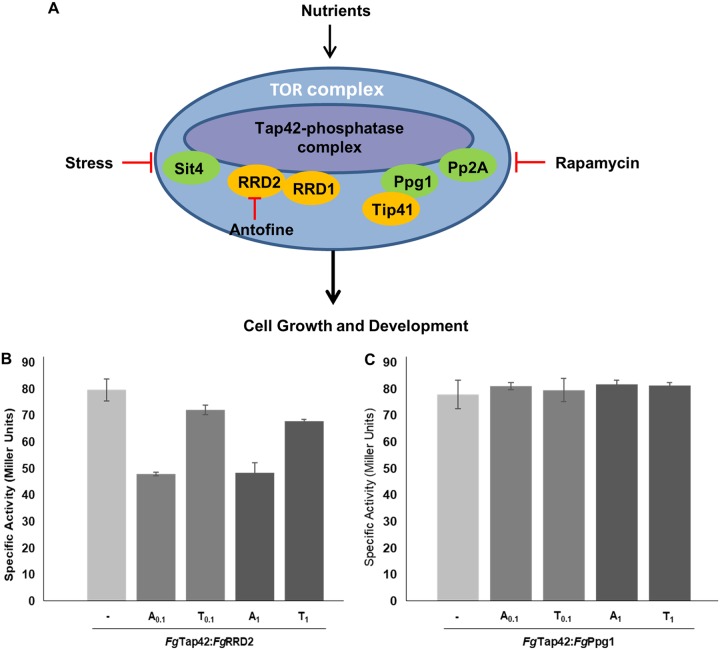
Antofine targets RRD2 and disrupts the Tap42-phosphatase complex in F. graminearum. (A) Model representing the TOR complex with components of the Tap42-phosphatase complex highlighting the negative regulation of RRD2 by antofine. The phosphatases are colored green. (B) Antofine specifically disrupts *Fg*Tap42-*Fg*RRD2 protein interaction in the Tap42 complex. The gene pairs *Fg*Tap42 and *Fg*RRD2 and *Fg*Tap42 and*Fg*Ppg1 were expressed in yeast in the absence (−) and in the presence of antofine and tylophorine at 0.1 and 1 μg/ml. The β-galactosidase specific activity (in Miller units) was measured by the ONPG assay (23). (C) The interaction between Tap42 and *Fg*Ppg1 has been demonstrated previously and was used as a positive control (11). Shown is a representative of three independent biological replicates, each with three technical replicates.

In F. graminearum, the interactions between components of the Tap42-phosphatase complex have been demonstrated (11, 13). Specifically, FGSG_09800 (Tap42) interacts with the phosphatases Sit4 (FGSG_01464), Ppg1 (FGSG_05281), and Pp2A (FGSG_09815), protein phosphatase activators RRD1 (FGSG_09229) and RRD2 (FGSG_01092), and a Tap42 interactor protein, Tip41 (FGSG_06963) (Fig. 3A) (11, 13). Mutation of the phosphatases Sit4 and Ppg1, the Tap42 interactor Tip41, or the phosphatase activators RRD1 and RRD2 leads to reduction of virulence of F. graminearum (11, 13). Since antofine appears to target RRD2 (FGSG_01092), we tested whether its interactions with Tap42 complex proteins are disrupted by antofine. We performed yeast two-hybrid (Y2H) analysis between *Fg*RRD2 and *Fg*Tap42, *Fg*Sit4, *Fg*Ppg1, *Fg*Pp2A, and *Fg*Tip41 and found that *Fg*RRD2 interacted only with *Fg*Tap42 (see Fig. S6A in the supplemental material). To validate these findings, we quantitatively assessed the interaction between *Fg*Tap42 and *Fg*RRD2 by ONPG (*o*-nitrophenyl-β-d-galactopyranoside) assay (Fig. 3B) (23). The strong interaction between Tap42 and RRD2 was inhibited even at a very low concentration of antofine (0.1 μg/ml [Fig. 3B]), while another interaction between Tap42 and Ppg1 remained intact even at higher concentrations of antofine (1 μg/ml [Fig. 3C]). Inhibition of the *Fg*Tap42-*Fg*RRD2 interaction was relatively specific to antofine (*P* < 0.0002) since inhibition with tylophorine was weak and only significant at the higher concentration tested (1 μg/ml [Fig. 3A]). Finally, we showed that antofine treatment was not deleterious to the accumulation of *Fg*RRD2, *Fg*Tap42, and *Fg*Ppg1 proteins (Fig. S6B). Since RRD binds to Tap42, we hypothesize that disruption of interaction between *Fg*RRD2 and Tap42 by antofine may deregulate the disassembly process of the Tap42-phosphatase complex with TORC1, potentially affecting Tor activation.

10.1128/mBio.00792-19.6 FIG S6 *Fg*RRD2 interacts with *Fg*Tap42. (A) A yeast two-hybrid analysis was performed between *Fg*RRD2 and *Fg*Tap42, *Fg*Ppg1, *Fg*Tip41, *Fg*Sit4, and *Fg*Pp2A. The interaction was performed with six independent transformants (indicated by a box). (B) The expression of *Fg*Tap42*, Fg*RRD2, and *Fg*Ppg1 in yeast was not affected by antofine treatment. An aliquot (20 μg) of protein was extracted from uninduced cultures expressing the gene pairs *Fg*Tap42/*Fg*RRD2 (lane 1)and *Fg*Tap42/*Fg*Ppg1 (lane 2) and from induced cultures expressing the gene pairs *Fg*Tap42/*Fg*RRD2 (lane 3) and *Fg*Tap42/*Fg*Ppg1 (lane 5) with no antofine and with 0.1 μg/ml antofine (lane 4) or tylophorine (lane 6).The proteins were separated by SDS-PAGE and transferred to nitrocellulose membrane, stained with Ponceau S, probed with LexA antibodies to detect *Fg*Tap42 (dashed arrowhead) and HA antibodies to detect both *Fg*RRD2 (filled arrowhead) and *Fg*Ppg1 (open arrowhead) proteins. The Western blots (WB) were detected by ECL substrate (Lumigen ECL Ultra, TMA-6). Download FIG S6, TIF file, 1.9 MB. © Crown copyright 2019. 2019 Crown This content is distributed under the terms of the Creative Commons Attribution 4.0 International license.

In summary, we have identified targets of antofine in F. graminearum, one of which (RRD2) is crucial for pathogenicity in wheat. Our finding that the plant-derived compound antofine directly impacts the action of TOR signaling provides an additional tool to dissect this complex signaling pathway and a potential lead compound for integrative pest management programs in crop protection. In addition to rapamycin, other inhibitors of the TOR pathway such as PP242, which acts as a competitive inhibitor for the ATP binding site of mammalian TOR, have been proposed for therapeutic use (24). More recently, PP242 was also used to inhibit the plant Tor kinase and reduce F. graminearum disease development in *Arabidopsis* (25). A similar increase in resistance to pathogens was observed when signaling from the TOR pathway was attenuated in *Arabidopsis* with a mutation in the *Raptor* gene, a direct target of the Tor kinase (26). However, the same study showed that manipulation of the TOR pathway could have unintended negative effects on plant growth, which presents an obvious hurdle to targeting this pathway for crop protection.

Large-scale fungicide use in agriculture is leading to emergence of multidrug resistance (MDR) in fungal strains, characterized by partial to complete resistance to fungicides (10). In this context, combinatorial drug application is one approach that can be used to combat MDR pathogens in agriculture. Such an approach is common in treating human diseases such as HIV and malaria and is also a strategy to combat deadly human fungal pathogens, such as *Candida*, *Aspergillus*, and *Cryptococcus*, and block the emergence of resistance (27, 28). The latter study showed that the small molecule beauvericin increased efficacy of azole-derived compounds through the inhibition of a multidrug efflux pump and the TOR signaling pathway (28). Similarly, a checkerboard analysis between antofine and existing fungicides could reveal concentrations of antofine that are benign to humans but can increase the efficacy of other commonly used azole-derived fungicides used in agriculture (29).

## MATERIALS AND METHODS

### Purification and confirmation by NMR of antofine.

Root tissues of Vincetoxium rossicum were harvested, vacuum desiccated, and crushed in EtOAc. The crude extract was filtered, concentrated by rotary evaporator, and dissolved in 0.01 M HCl. The pH was reduced to 2.0 with 1 M HCl and the solution partitioned with EtOAc. The organic fraction was concentrated by rotary evaporator and dried for storage. The pH of the aqueous fraction was increased to 10 with 1 M NH_4_OH and partitioned with EtOAc. The alkaline fraction containing antofine was concentrated by rotary evaporator, dissolved in pure acetonitrile (ACN), and injected into a 250- by 10-mm 5-μm 120-Å C_18_ reverse-phase semipreparatory column (ACE-121-2510; Advanced Chromatography Technologies). The column was preequilibrated in a gradient (5% ACN in water to 75% ACN in water) over 20 min at 10 ml/min. Antofine was eluted in 75% ACN–distilled water (dH_2_O) in 10-ml fractions for 20 min. An aliquot of 2 ml from each sample was dried using a SpeedVac (SVC-100H; Savant) and suspended in 200 μl methanol (MeOH). A 10-μl aliquot was spotted onto a sterile 6-mm filter disc. The discs were placed amended side down on petri dishes containing 2% YPD (yeast extract-peptone-dextrose) agar evenly spread with ∼1.0 × 10^6^ CFU of S. cerevisiae BY4741 and incubated at 30°C overnight. The fractions that demonstrated an inhibition zone were pooled and dried using a rotary evaporator. The pellet was suspended in MeOH and spotted onto a glass-backed silica thin-layer chromatography (TLC) plate (5715-7; EMD Chemicals). Compounds were separated by TLC with two consecutive migrations of 60:40 EtOAc-MeOH and traced by illumination under shortwave (254-nm) UV light. Each band was scraped from the silica plate and recovered using MeOH. The MeOH was removed by rotary evaporator, and the recovered compounds were solubilized in CHCl_3_ and tested for activity as described before. Purified antofine was subjected to ^1^H nuclear magnetic resonance (NMR) for confirmation. The NMR spectrum was recorded on a Bruker Avance 400-MHz NMR spectrometer. The sample was recorded using CDCl_3_ as the solvent, purchased from Cambridge Isotope Laboratories, Inc. The reference peak was set using the known residual solvent peak of CDCl_3_ at 7.26 ppm. The NMR spectrum was compared to the known spectrum of antofine (30).

### Identification of antofine targets in yeast by haploinsufficiency profiling.

The Yeast Heterozygous Knock-Out collection (Open Biosystems; GE Healthcare, USA), along with the diploid wild-type strain (BY4743), was copied to solid 2% YPD agar amended with 150 μg/ml G418, contained in an Omniwell plate (Nunc catalog no. 242811), using a 96-pin replicator (Nunc catalog no. 250520) and the OmniTray copier (Nunc catalog no. 250555) and incubated overnight at 30°C. The initial screen was initiated with antofine (0.75 μg/ml) and incubated for 24 h at 30°C. The plates were scanned as a grayscale image (Epson Perfection V750 Pro) and processed with the Epson Scan version 3.24A scanning software at a 300-dpi resolution. The initial screen was repeated 6 times at antofine concentrations of 2.75, 2.5, 2.0, and 1.25 μg/ml, with a final concentration of methanol at 0.01%. The results of the initial screens were analyzed together using the Screen Mill analysis package, and all mutants that received a *P* value of <0.05 were selected for further analysis (31).

Our initial screen yielded 873 potential candidates. To further narrow the list of candidates, we employed a liquid-based analysis. Briefly, each mutant was grown to an absorbance at 600 nm (ABS_600_) of ∼5.0, and 10 μl of this new cell suspension was transferred to a new 96-well plate containing 190 μl of YPD and bringing the cell density equivalent to an ABS_600_ reading of 0.25 (∼5.0 × 10^6^ CFU/ml), supplemented with 0.20 μg/ml of Antofine. The plate was then loaded into a BioTek Powerwave plate reader, for incubation at 30°C, and then the growth was assessed by ABS_600_ every 10 min over the course of 48 h. The growth for each mutant (with antofine) was then compared to the average growth calculated for the WT (with antofine), by subtracting the calculated percentage of WT growth from the calculated percentage of growth for the mutant, where a positive result signified the mutant grew better than the WT under experimental conditions, and a negative result signified the mutant grew poorer than the WT when antofine was present. We used 10% as the cutoff for growth differences. This analysis yielded 16 mutants sensitive to antofine.

To confirm the observed effect of antofine, 16 antofine-sensitive mutants were further screened with three concentrations of antofine (2.5, 2, and 1.5 μg/ml) in triplicate. This was repeated three times. After analysis, three yeast mutants (YBR095C, YOR375C, and YIL153W) showed a consistent sensitive phenotype to antofine.

### ScreenMill analysis.

The differences in colony growth were assessed by the ScreenMill growth measurement and analysis software suite (31). Briefly, each plate is scanned and then analyzed using the colony measurement engine, which detects each colony and assigns each a numerical value based upon its circularity and size. The file output is then input into the data review engine. A web-based application normalizes raw screen data and provides the user with an opportunity to manually select data for exclusion from analysis. The normalized data are used to calculate *P* values by *t* test for parametric data or the Mann-Whitney test for nonparametric data.

### Construction of F. graminearum *Fggdh1* and *Fgrrd2* mutant strains and the *Fgrrd2*::OE*FgRRD2* overexpression strain.

F. graminearum wild-type strain DAOM 233423 (NRRL29169) was provided by C. Babcock of the Canadian Collection of Fungal Cultures (CCFC/DAOM), Agriculture and Agri-Food Canada, Ottawa. Macroconidia were used as the inoculum for all experiments and were produced in a liquid carboxymethyl cellulose medium (32). All of the plasmids were constructed by the USER (uracil-specific excision reagent) technique and outlined (see Fig. S7 in the supplemental material) (33). The deletion of *FgGDH* (FGSG*_*07174) and *FgRRD2* (FGSG_01092) was performed by PCR amplification of the two homologous recombination sequences (HRSs) for each gene. HRS1 for FGSG_07174 was obtained with primer sets Fg 07174 USER Up-F/R, and HRS1 for FGSG_01092 was obtained with Fg 01092 USER Up-F/R. HRS2 for FGSG_07174 was obtained with primer sets Fg 07174 USER Dn-F/R, and HRS2 for FGSG_01092 was obtained with Fg 01092 USER Dn-F/R. PCR products HRS1 and HRS2 were introduced into the pRF-HU2 vector with hygromycin (Hyg) as the selection marker. The *Fgrrd2*::OE*FgRRD2* overexpression strain was constructed by introducing FGSG_01092 cDNA into the pRF-GUE vector with Geneticin as the selection marker (Gen). The cDNA was amplified by the primer set Fg 01092 GUE_F/R. The F. graminearum mutants were screened for the presence and absence of FGSG_07174 and FGSG_01092 with primer sets Fg 07174 Orf F/R and Fg 01092 Orf F/R, respectively. The presence of the *gpdA* promoter in the *Fgrrd1*::OE*Fg RRD1*strain was confirmed by the primer set Gpd Pro F and Fg 01092 Orf R. The sequences of the primers are listed in Table S1 in the supplemental material. Touchdown PCR conditions were used to confirm F. graminearum transgenic strains: 100 ng of genomic DNA for 8 cycles at 63 to 55°C and for 22 cycles at 55°C. We verified the copy number in the mutant strains by quantitative real-time PCR (qRT-PCR) analyses (Fig. S7G). Briefly, the analyses were performed with the primer set Fg 01092 qPCR F/R for FGSG_01092 and the primer set Fg 07174 F/R for FGSG_07174 and normalized with a known single-copy gene coding for glyceraldehyde-3-phosphate dehydrogenase, *GAPDH* (FGSG_16627), using the primer set Fg 16627 F/R (Table S1) (34). All samples were assessed in triplicate using the QuantStudio3 (Applied Biosystems, USA).

10.1128/mBio.00792-19.7 FIG S7 Targeted deletion of *FgGDH1* and *FgRRD2* and overexpression of *FgRRD2* in F. graminearum. (A) A schematic representation of plasmid constructs used to delete *FgGDH1* (FGSG*_*07174) and *FgRRD2* (FGSG*_*01092). The two flanking regions surrounding the gene (HRS1 and HRS2) were cloned into pRF-HU2 vector and transformed into the wild-type F. graminearum strain using *Agrobacterium*-mediated transformation and selected with the resistance marker hygromycin (Hyg). (B) A schematic representation of the plasmid construct used to overexpress *FgRRD2* (FGSG*_*01092). A cDNA corresponding to FGSG_01092 was cloned into the vector pRF-GUE and transformed into the F. graminearum ΔFGSG_01092 mutant strain using *Agrobacterium*-mediated transformation and selected with the resistance marker geneticin (Gen). (C) PCR confirmation of deletion of *FgGDH1*. Genomic DNAs from the wild-type (lane 2) and the ΔFGSG_07174 mutant (lane 3) F. graminearum strains were screened for the presence of the FGSG*_*07174 gene with Fg07174 Orf F/R primers (Fg07174), the FGSG_01092 gene with Fg09192 Orf F/R primers (Fg01092), and the resistance marker hygromycin (Hyg) with Hyg F/R primers (Table S1). Lane 1 represents the water control. (D) PCR confirmation of deletion of *FgRRD1*. Genomic DNAs from the wild-type (lane 2) and the ΔFGSG_09192 (lane 3) F. graminearum strains were screened for the presence of the FGSG*_*07174 gene with Fg07174 Orf F/R primers (Fg07174), the FGSG*_*01092 gene with Fg09192 Orf F/R primers (Fg01092), and the resistance marker hygromycin (Hyg) with Hyg F/R primers (Table S1). Lane 1 represents the water control. (E) PCR confirmation of overexpression of *FgRRD2* in the ΔFGSG*_*01092 strain (*Fgrrd2*::OE*FgRRD2*). Genomic DNAs from the wild type (lane 2) and the *Fgrrd2*::OE*Fg RRD2* overexpression strain (lane 3) were screened for the presence of the resistance marker hygromycin (Hyg) with Hyg F/R primers, the resistance marker geneticin (Gen) with Gen F/R primers, and the presence of FGSG*_*01092 cDNA with the primer set pGPD Pro F and FGSG*_*01092 Orf R (pGPD-Fg01092). Lane 1 represents the water control. (F) RT-PCR confirmation of expression of *FgRRD1*. cDNA was synthesized from total RNA isolated from the F. graminearum wild-type strain (lanes 1 and 2), the ΔFGSG_09192 deletion strain (lanes 3 and 4), and the *Fgrrd1*::OE*FgRRD2* overexpression strain (lanes 5 and 6). PCR was used to screen for the presence of the FGSG*_*01092 gene with Fg09192 Orf F/R primers (Fg01092) and an internal control gene, *TRI10*, with the Tri10 F/R primer set (Tri10) (Table S1). Lanes 1, 3, and 5 represent the water control. (G) Copy number assessment of the FGSG*_*07174 and FGSG*_*01092 genes in the ΔFGSG_07174 and ΔFGSG*_*01092 mutant strains, respectively, by qRT-PCR analyses. Download FIG S7, TIF file, 0.8 MB. © Crown copyright 2019. 2019 Crown This content is distributed under the terms of the Creative Commons Attribution 4.0 International license.

10.1128/mBio.00792-19.8 TABLE S1 List of primers used in this study. Download Table S1, DOCX file, 0.1 MB. © Crown copyright 2019. 2019 Crown This content is distributed under the terms of the Creative Commons Attribution 4.0 International license.

### RNA extraction and cDNA library from F. graminearum.

F. graminearum strains were grown in a mixture of 56 mM NH_4_Cl, 8.1 mM MgSO_4_·7H_2_O, 0.23 mM FeSO_4_·7H_2_O, 14.7 mM KH_2_PO_4_, 2 g/liter peptone, 2 g/liter yeast extract, 2 g/liter malt extract, and 111 mM glucose for 24 h, and total RNA was extracted from mycelia using TRIzol reagent (Invitrogen), purified using the InviTrap Spin cell RNA minikit (Stratec, Germany), and then converted into cDNA (1 μg) using the Applied Biosystems high-capacity cDNA reverse transcription kit. RT-PCRs were similar to the touchdown PCR conditions mentioned before.

### Growth curve assays with inhibitors.

One thousand spores from F. graminearum wild-type (*Wt*), ΔFGSG 07174 and ΔFGSG 01092 mutant strains, and the *Fgrrd2*::OE*FgRRD2*strain were inoculated in 200 μl GYEP medium (0.1% KH_2_PO_4_, 0.05% MgSO_4_·7H_2_0, 0.05% KCl, 0.02% [vol/vol] trace element solution, and 0.01% sucrose). The spore germination assay was set up in 96-well microtiter plates (Corning 3632), and growth was monitored over a 48-h period at 600 nm using the FLUOstar Optima plate reader (BMG Labtech, USA) in a final volume of 200 μl. We assessed the germination and growth of various numbers of spores (250, 500, and 1,000) by absorbance (λ = 600 nm) and visualization at numerous time points with a Zeiss AxioImager M2 microscope (Carl Zeiss Canada, Toronto, Ontario) (Fig. S1B). An absorbance reading of ∼0.5 was the same for all the spore concentrations at 48 h. Therefore, we used 48 h as the endpoint measurement for all of the fusarium spore germination/growth assays. All yeast strains were grown overnight in YPD (yeast extract-peptone-dextrose) at 30°C with shaking at 200 rpm overnight. Cells were diluted to an ABS_600_ of 0.5 and used in growth curve assays. The growth was monitored over a 24-h period at 600 nm using the FLUOstar Optima plate reader as described before. The inhibitors were dissolved in 100% methanol and were used at the indicated concentrations in a 1% final concentration of methanol in all of the liquid assays.

### Purification of *Fg*GDH (FGSG_07174) and *Fg*RRD1 (FGSG_01092) from yeast.

Both FGSG_07174 and FGSG_01092 were cloned into the pYES-DEST 52 Gateway destination plasmid vector by LR clonase reaction (Life Technology, USA). Plasmids were transformed into yeast knockout strains YOR375C and YIL153W, respectively. The complemented yeast was grown in 5 ml of Sabouraud dextrose (SD) medium lacking uracil in the presence of 2% glucose and antibiotic selection with G418 (Sigma) overnight at 30°C. Yeast cells were transferred in a 1:100 ratio to 30 ml of inducing medium with 2% galactose, 1% raffinose, and G418 overnight at 30°C. Yeast cells then were harvested by centrifugation at 2,700 × *g* for 10 min. The resulting yeast pellet was processed for protein extraction. The His-tagged proteins were purified using the Clontech His-tagged purification miniprep kit. Yeast pellet containing the His-tagged protein was thawed in 1 ml X-tractor buffer, 100 μl Zymolyase (5 U/μl; G Biosciences), 40 μl of 25× Roche Complete mini-protease inhibitor cocktail, 10 μl RNase A (20 mg/ml; Sigma), and 5 μl DNase I (10 U/μl; Agilent). The lysate was incubated at 37°C for 1 h and purified according to the manufacturer’s instructions with modifications. Briefly, the total lysates were centrifuged at 15,000 rpm to get rid of the cellular debris, and the supernatant was loaded onto the affinity column. After washes with buffer (20 mM Tris [pH 8.0], 30 mM NaCl, 2 mM dithiothreitol [DTT], and protease inhibitor cocktail) containing 20 and 50 mM imidazole, proteins were eluted with buffer containing 500 mM imidazole. Eluates were dialyzed with buffer overnight at 4°C. Glycerol was added to a final concentration of 20%, and the mixture was stored at −20°C until use.

### TSAs.

Thermal shift assays (TSAs) were performed with purified *Fg*GDH and *Fg*RRD2 proteins. The TSA was performed in Quant Studio 3 (Applied Biosystems) with the Applied Biosystems protein thermal shift dye kit (4461146) in a 20-μl reaction volume containing 1 mg/ml of each protein with or without antofine or tylophorine (Santa Cruz Biotechnologies, USA) at 100 μg/ml (0.1% methanol). The PCR conditions were used according to the manufacturer’s instructions, and data were analyzed with Protein Thermal Shift software version 1.3 (Thermo Fisher Scientific). Melting temperature (*T_m_*) data were generated using the Boltzmann method. Δ*T_m_* was calculated by comparing the *T_m_* values for each protein without the inhibitors to those with the inhibitors. Data were collected at 1°C intervals from 25°C through 99°C and are shown in the replicate result plot view of the Protein Thermal Shift software.

### *Fusarium* infection assay.

Each strain was assessed for the ability to infect susceptible Triticum aestivum (cv. Roblin). Wheat heads (10 heads per strain) were point inoculated at mid-anthesis with each strain at 100,000 spores/ml in a volume of 10 μl (1,000 spores). Spores were inoculated between the palea and lemma of a single wheat spikelet. Plants were grown at 25°C for 16-h days with misting every hour for 30 s. After 48 h, misting was reduced to a duration of 30 s every 4 h. Infection was scored by counting the number of visibly infected spikelets at days 5, 10, and 15 (32).

### Yeast two-hybrid and ONPG assays.

The coding sequences of *Fg*Tap42, *Fg*RRD2, *Fg*Tip41, *Fg*Pp2A, and *Fg*Ppg1 from F. graminearum were synthesized and cloned into the pDONR vector (General Biosystems, Morrisville, NC). The genes were subcloned into the yeast vectors pJG4-5 (activation domain B42) and pEG202 (DNA binding domain LexA) by Gateway LR Clonase II reaction (Thermo Fisher Scientific). Yeast two-hybrid plasmid pairs were transformed into S. cerevisiae strains RFY206 and EGY48, before mating and selection on synthetic medium lacking histidine, uracil, and tryptophan. Two independent yeast colonies containing both pJG4-5 and pEG202 for each gene pair were selected for interaction, and the interaction was quantified by a β-galactosidase activity assay with ONPG (23). Interaction was monitored in the presence of either antofine or tylophorine (0, 0.1, and 1 μg/ml). Three independent experiments were performed, using corresponding empty vectors as negative controls. All the yeast strains were grown as described before in the purification of *Fg*GDH1 and *Fg*RRD1, except the induction with galactose was limited to 1 h. To ensure that expressed proteins were not affected by antofine, extracts from noninduced and induced cultures (±antofine or tylophorine) were subjected to Western blot analyses. Proteins were extracted by 2 M LiAc and 0.4 M NaOH followed by boiling in Laemmli buffer. Twenty micrograms of protein from each sample was separated on a 10% SDS-PAGE gel, and the blots were probed with LexA antibodies for detection of *Fg*Tap42 (Millipore Sigma, USA) and hemagglutinin (HA) antibodies (Roche, Germany) to detect both *Fg*RRD2 and *Fg*Ppg1 proteins. Proteins were detected by the ECL enhanced chemiluminescence substrate (Lumigen ECL Ultra; TMA-6).
