# Supplementary figures and images for: Genomic Identification of the TOR Signaling Pathway as a Target of the Plant Alkaloid Antofine in the Phytopathogen Fusarium graminearum
Source: mBio. 2019 Jun 11;10(3):e00792-19. doi: 10.1128/mBio.00792-19 (PMC6561021; doi:10.1128/mBio.00792-19)

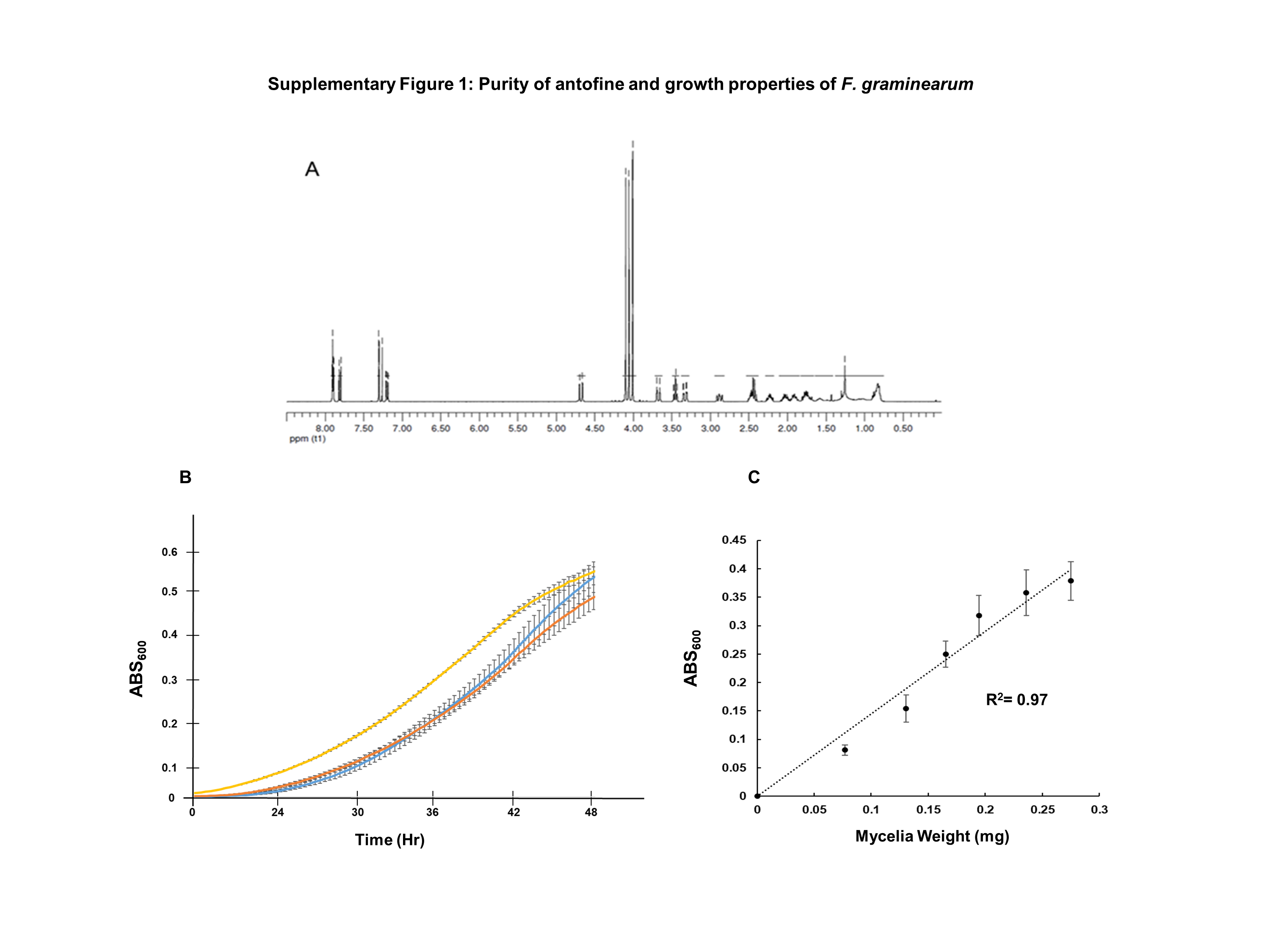

Supplement: FIG S1 [file mBio.00792-19-sf001.tif]

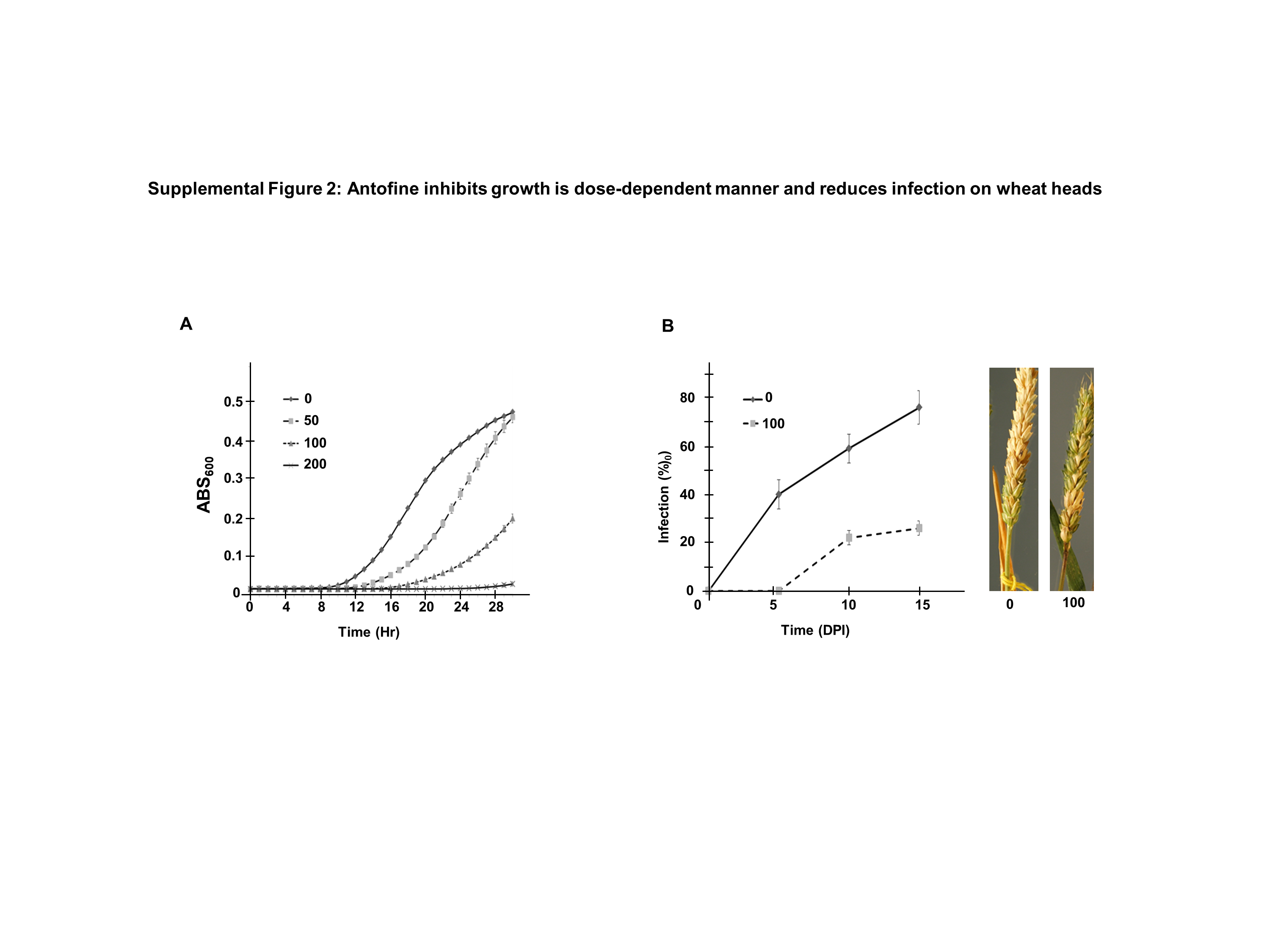

Supplement: FIG S2 [file mBio.00792-19-sf002.tif]

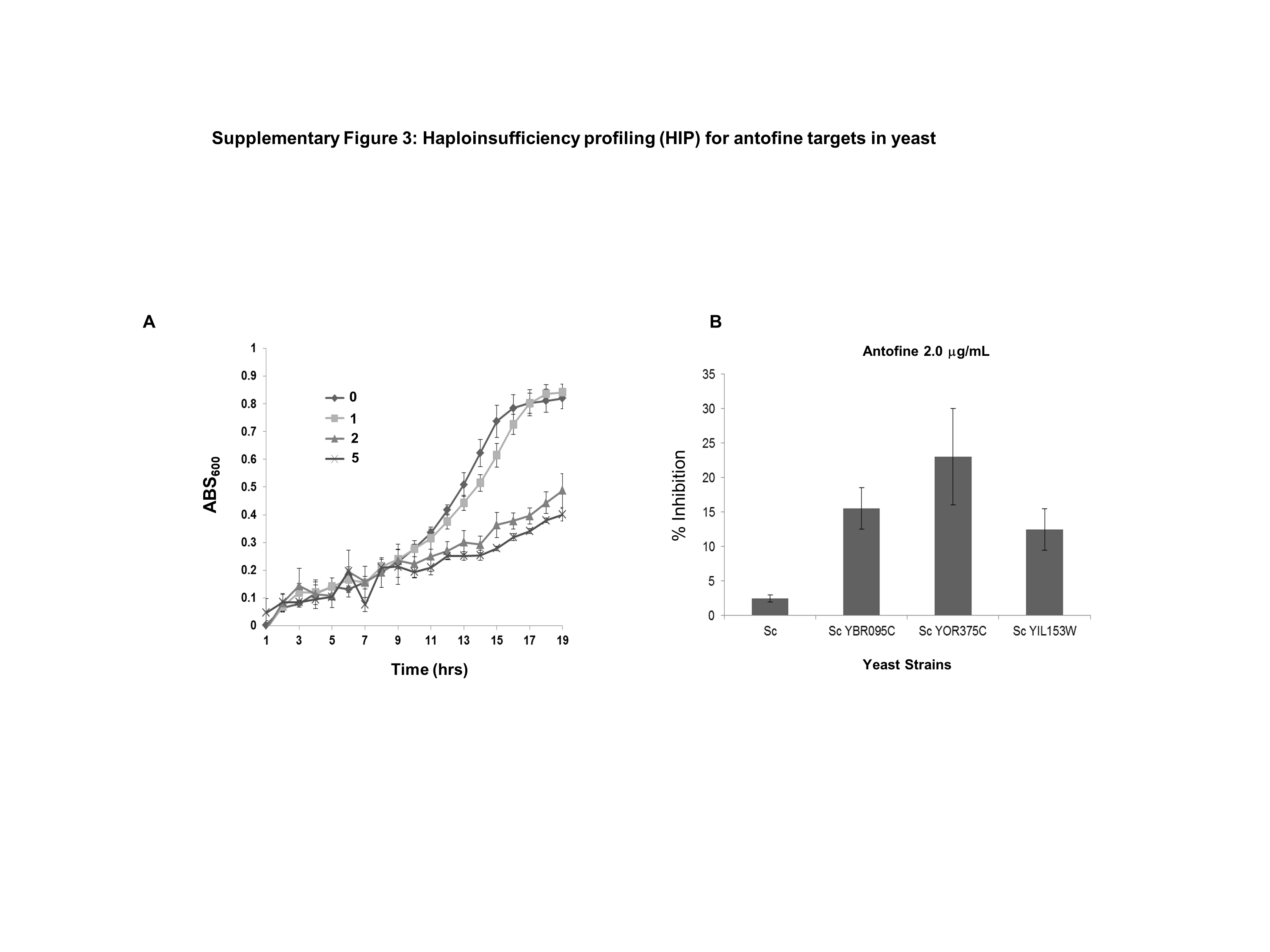

Supplement: FIG S3 [file mBio.00792-19-sf003.tif]

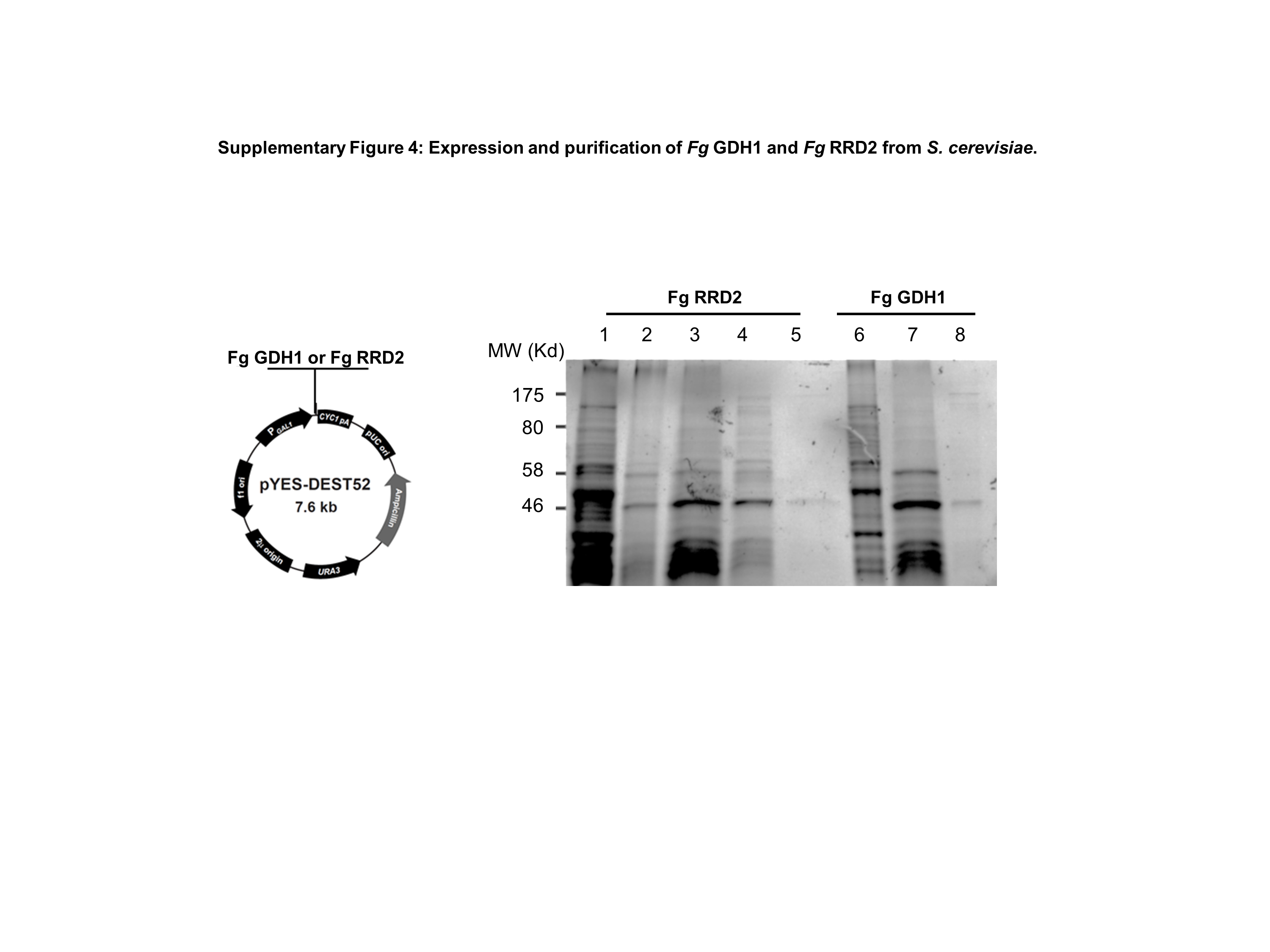

Supplement: FIG S4 [file mBio.00792-19-sf004.tif]

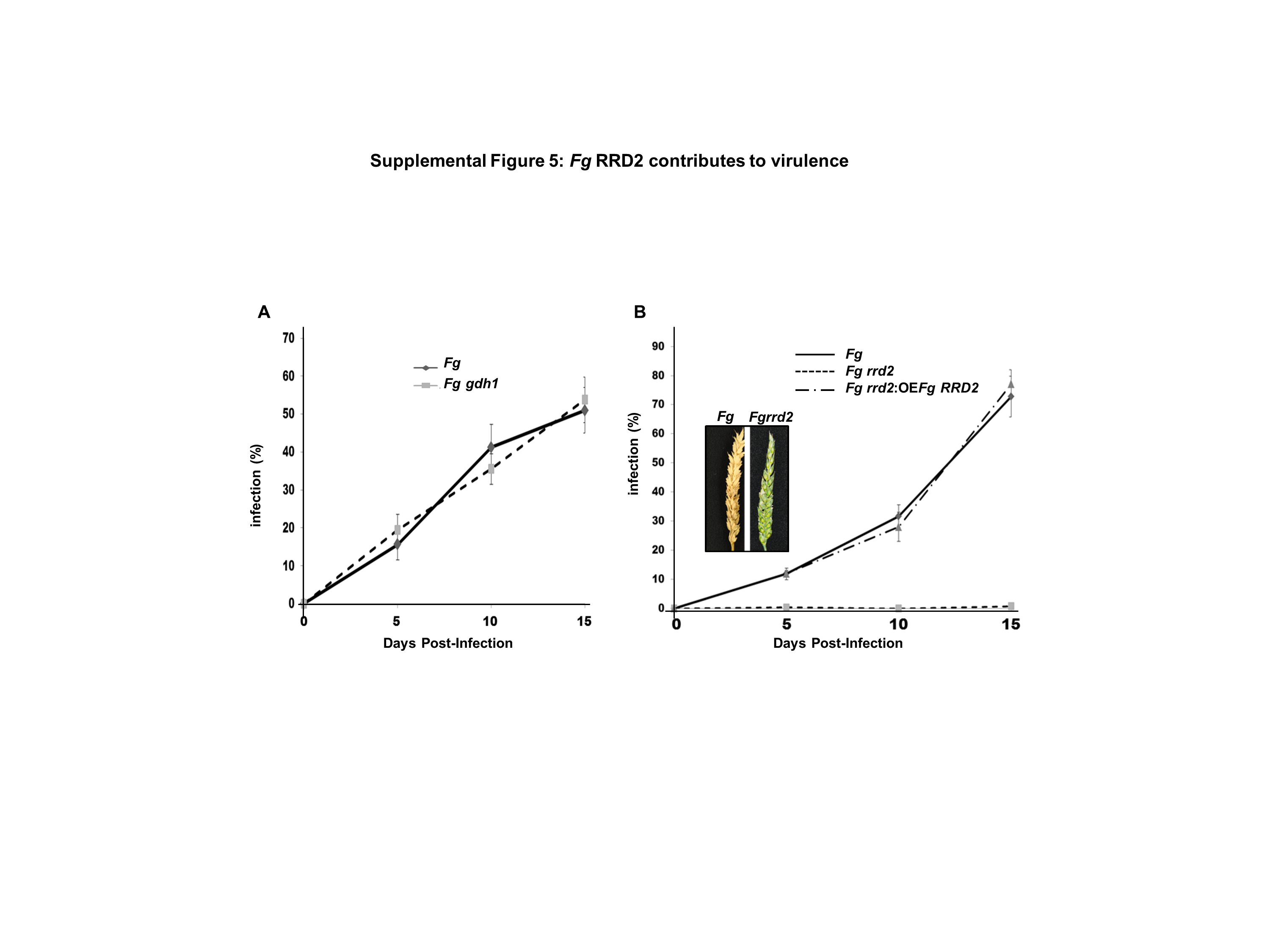

Supplement: FIG S5 [file mBio.00792-19-sf005.tif]

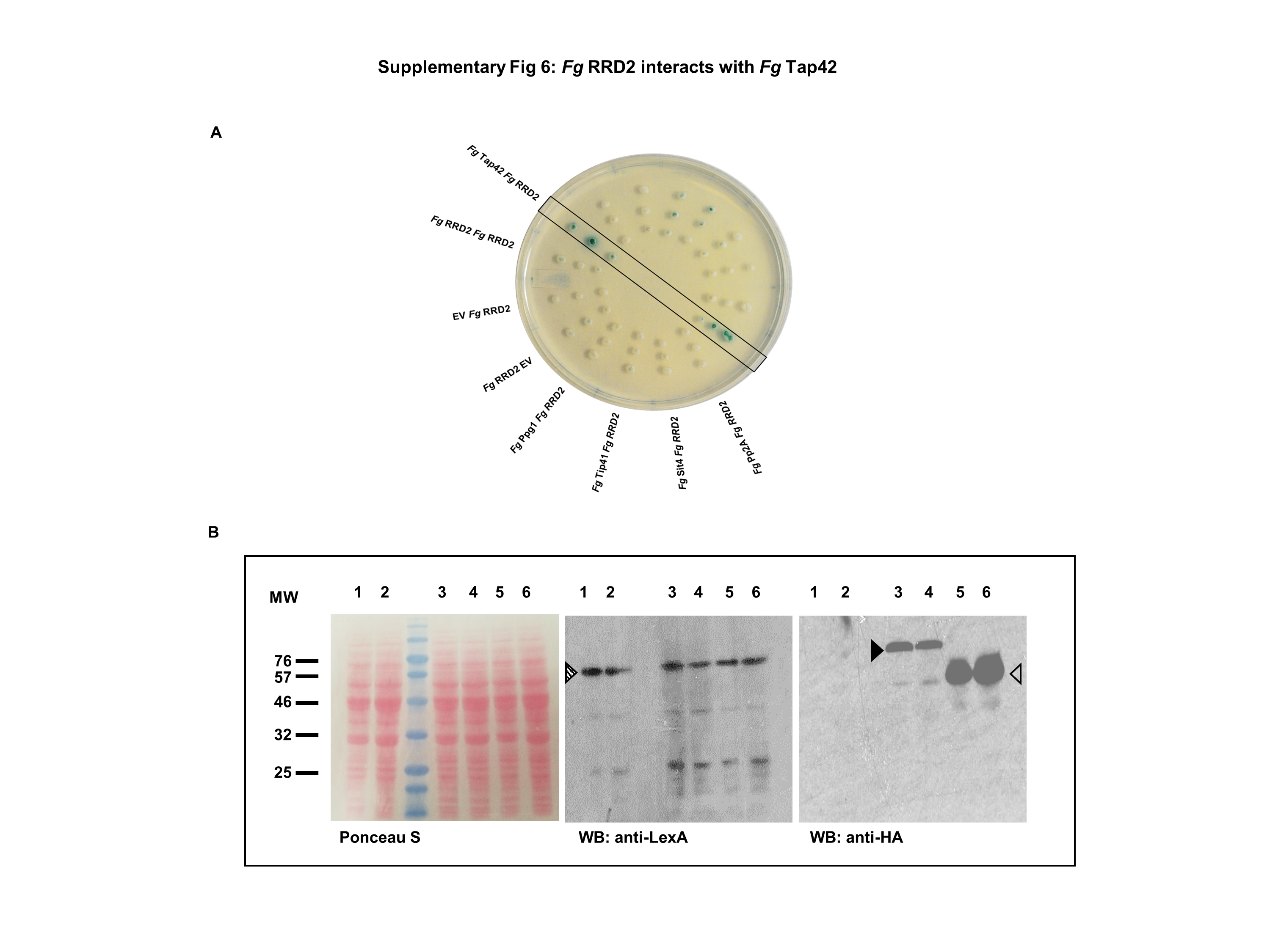

Supplement: FIG S6 [file mBio.00792-19-sf006.tif]

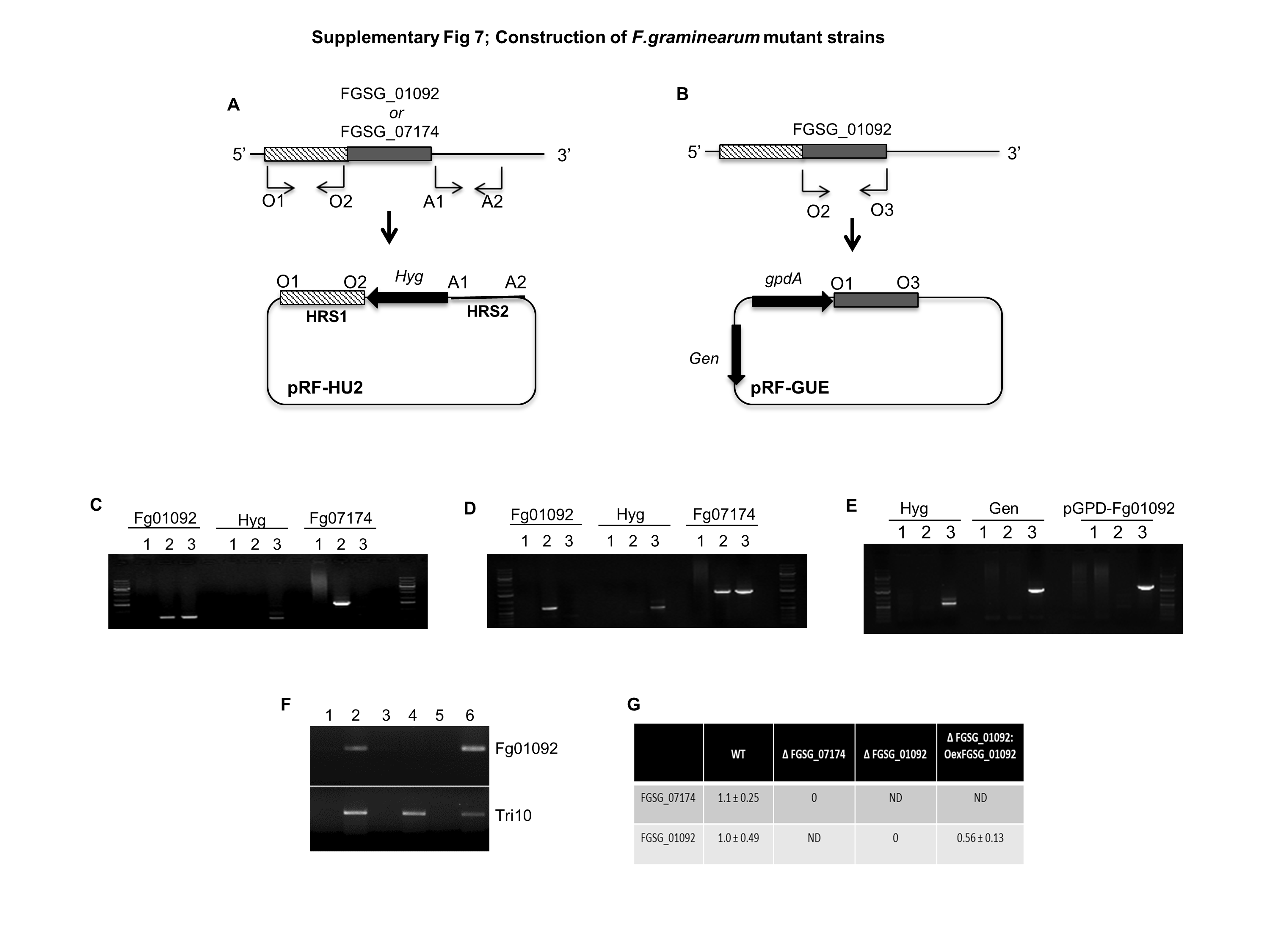

Supplement: FIG S7 [file mBio.00792-19-sf007.tif]
