# Supplementary material for: Genomic Identification of the TOR Signaling Pathway as a Target of the Plant Alkaloid Antofine in the Phytopathogen Fusarium graminearum
Source: mBio. 2019 Jun 11;10(3):e00792-19. doi: 10.1128/mBio.00792-19 (PMC6561021; doi:10.1128/mBio.00792-19)
Supplement: TABLE S1 [file mBio.00792-19-st001.docx]

**Supplementary Table 1**

| **Primer name** | **Sequence 5' – 3'** |
| --- | --- |
| Fg 07174 USER Up F | GGGTTTAUGGTGTATTGTTAGTGGTC |
| Fg 07174 USER Up R | GGACTTAUGTCGATGTCTGGTTGGAG |
| Fg 07174 USER Dn F | GGCATTAUGACGAGAGATCCGAGACT |
| Fg 07174 USER Dn R | GGTCTTAUTCCTGGTATAAGTGTGGC |
| Fg 07174 Orf F | CTGGTAAGGGTCTCTCTTGGGGT |
| Fg 07174 Orf R | TCTCAAGACCAGAAACGGCAACAC |
| Fg 01092 USER Up F | GGGTTTAUGTGTTGAAACGACTAGGT |
| Fg 01092 USER Up R | GAACTTAUATGGTCCTTCTTGGACAG |
| Fg 01092 USER Dn F | GGCATTAUCGACATAGGCTTTGATGC |
| Fg 01092 USER Dn R | GGTCTTAUCAATCAAGATCTTCCGAC |
| Fg 01092 Orf_F | ATGACTTCACAGGCGCCC |
| Fg 01092 OrF_R | TTAATCGAATGGGATTCTTCGC |
| Fg 01092 GUE_F | GGACTTAAUATGACTTCACAGGCGCCC |
| Fg 01092 GUE_R | GGGTTTAAUTTAATCGAATGGGATTCTTCGC |
| Gpd Pro F | GAGCTCTGTACAGTGACC |
| Hyg F | AGCTGCGCCGATGGTTTCTACAA |
| Hyg R | GCGCGTCTGCTGCTCCATACAA |
| Gen F | TCATCAATCCCAGCCTTTTC |
| Gen R | CAGTCGATGAATCCAGAAAAGC |
| Tri10 F | GACGCTTCAATTGTTG |
| Tri10 R | CAACAATTGAAGCGTC |
| Fg 01092 qPCR F | CATCCTCGACGAGACAGAGC |
| Fg 01092 qPCR R | TACGCCGATATCATGGTGCC |
| Fg 07174 qPCR F | CACCGGTTACGGTCTTGTCT |
| Fg 07174 qPCR R | GCTCGATGATCTTGAGGGCA |
| Fg 16627 qPCR F | TGACTTGACTGTTCGCCTCGAGAA |
| Fg 16627 qPCR F | ATGGAGGAGTTGGTGTTGCCGTTA |
|  |  |
|  |  |
|  |  |
|  |  |
|  |  |
|  |  |
